# Supplementary material for: MTAP deficiency creates an exploitable target for antifolate therapy in 9p21-loss cancers
Source: Nat Commun. 2022 Apr 4;13:1797. doi: 10.1038/s41467-022-29397-z (PMC8980015; doi:10.1038/s41467-022-29397-z)
Supplement: Supplementary file 1 — Supplementary Information [file 41467_2022_29397_MOESM1_ESM.pdf]

Inventory of Supporting Information

- Supplementary Figures and Legend
- Supplementary Tables and Legend

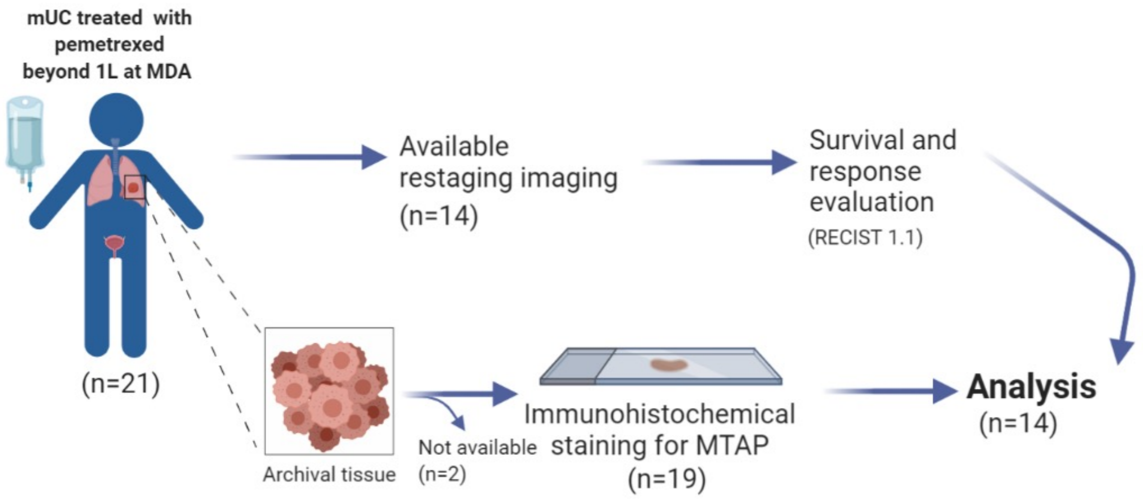

Supplementary Fig. 1. Schema of retrospective analysis. MTAP^{def}, MTAP deficient; MTAP^{prof}, MTAP proficient. Schema to demonstrate the retrospective analysis performed on patients with metastatic urothelial carcinoma and archival tissue available for MTAP immunohistochemical staining to correlate with response to pemetrexed.

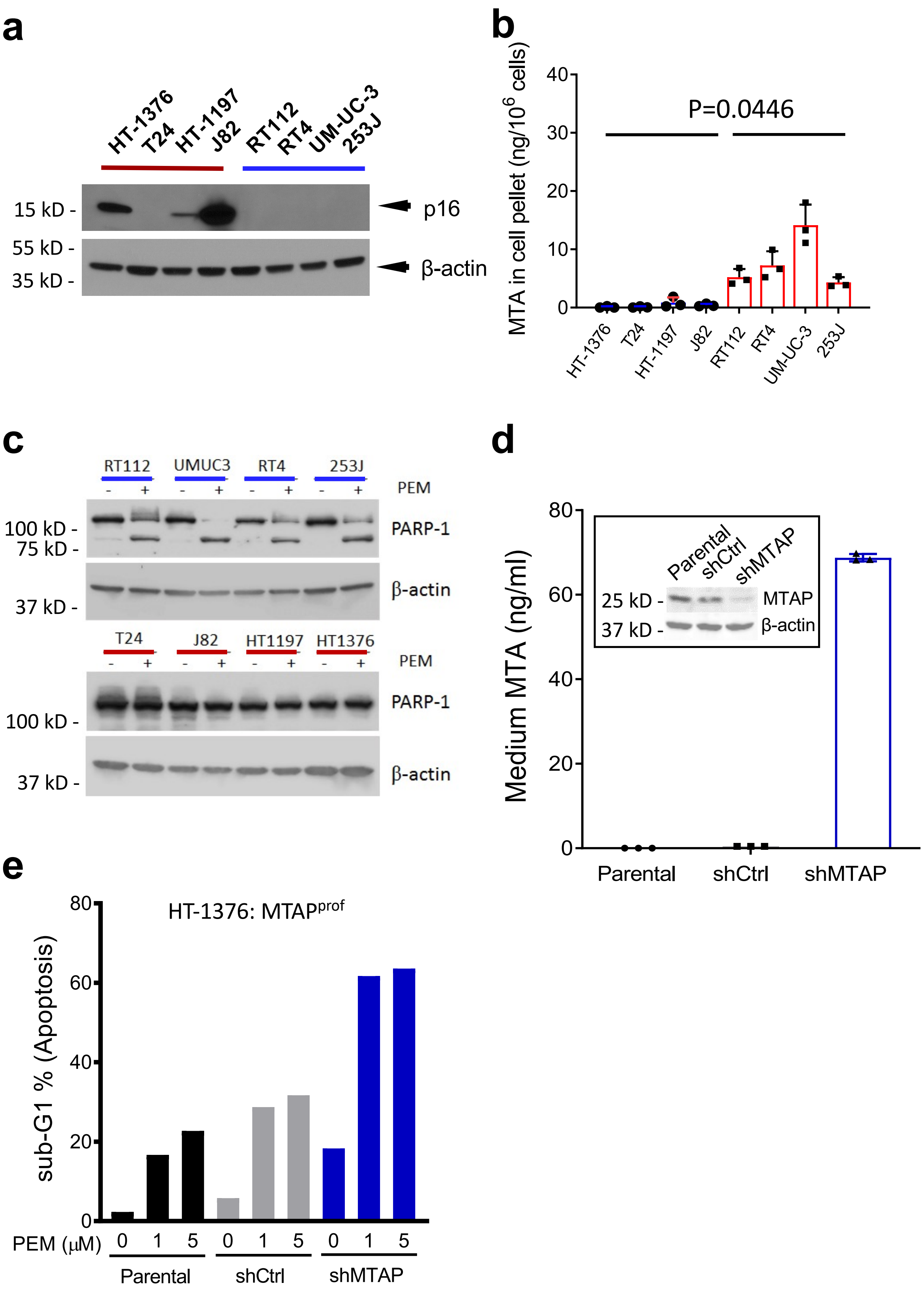

Supplementary Fig. 2. ***In vitro*** **analysis of functional loss of MTAP and apoptotic response to pemetrexed assessment.** MTAP^{def}, MTAP deficient; MTAP^{prof}, MTAP proficient. **(a)** p16 protein levels in eight human bladder carcinoma cell lines. **(b)** Cell culture pellet MTA level between MTAP^{def} and MTAP^{prof} cell lines. P value was calculated by Welch's T test. **(c)** Pemetrexed treatment can induce significant PARP1 cleavage in MTAP^{def} cell lines but not in MTAP^{prof} cell lines. **(d)** MTA levels increased after *MTAP* knockdown with shRNAs in HT1376 cell line. In both (b) and (d) , n=3 biologically independent samples for each cell line were used. Data in b and d (bottom panel) are presented as mean +/- SD. **(e)** *MTAP* knockdown in HT1376 cell line increased sensitivity to pemetrexed treatment.

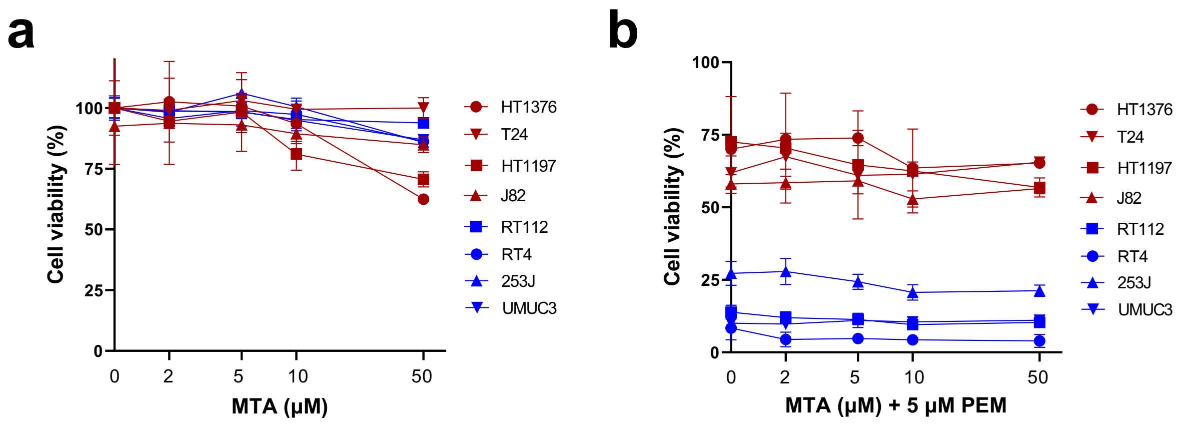

Supplementary Fig. 3. Effect of MTA alone or combined with pemetrexed on cell viability among UC cell lines. (a) both MTAP^{prof} (red) and MTAP^{def} (blue) UC cell lines are resistant to MTA therapy. **(b)** the increased sensitivity of MTAP^{def} (blue) as compared to MTAP^{prof} (red) UC cell lines upon exposure to pemetrexed (5 ul) is not altered with increasing doses of MTA. n=3 biologically independent samples for each cell line. Data are presented as mean +/- SD.

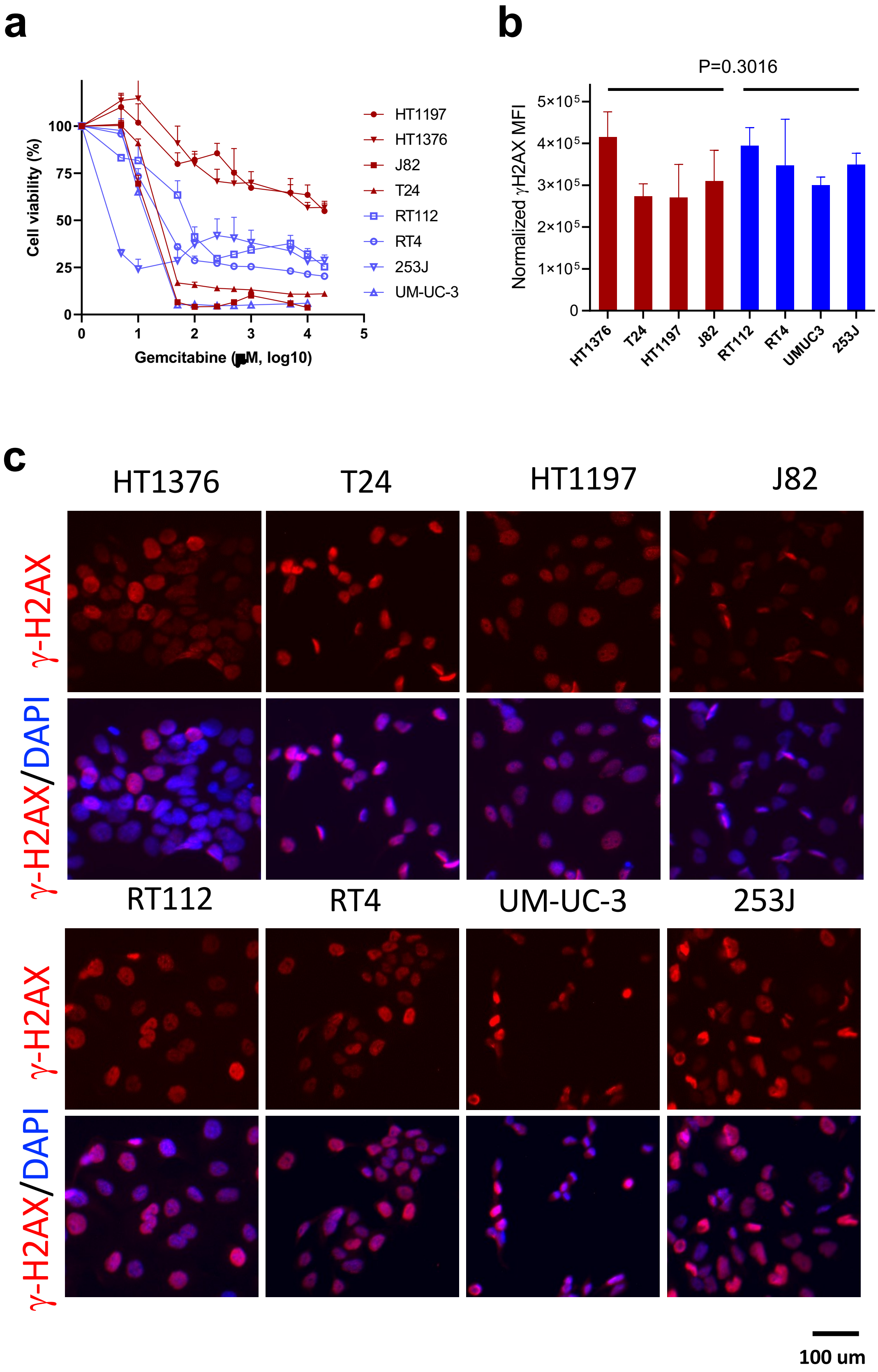

Supplementary Fig. 4. Gemcitabine induces undifferentiated cytotoxicity and DNA damage regardless of MTAP status in vitro. **(a)** Gemcitabine treatment for 72h lead to cell growth inhibition in both MTAP^{def} and MTAP^{prof} bladder cancer cell lines in vitro. n=4 biologically independent samples for each cell line **(b)** g-H2AX staining showed similar effects on DNA damage were induced in both MTAP^{def} and MTAP^{prof} cells treated with 1 μM gemcitabine for 24h. n=5 biologically independent samples for each cell. Data are presented as mean +/- SEM. P value was calculated using Welch T test. **(c)** Representative images for g-H2AX (red) and DAPI (blue) immunofluorescence staining in eight bladder cancer cell lines.

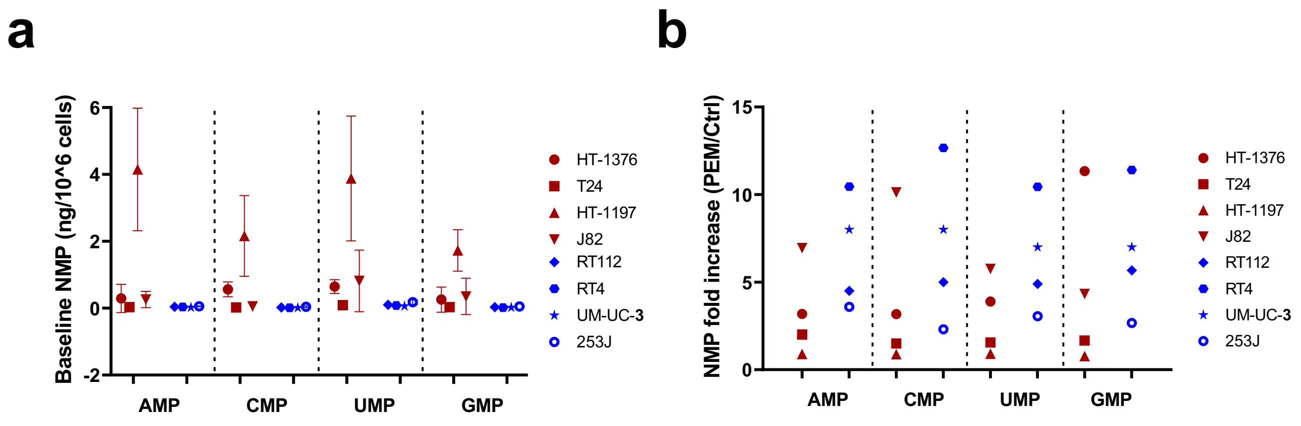

Supplementary Fig. 5. Distorted nucleotide monophosphate (NMP) pools *in vitro* upon therapy with pemetrexed. (a) baseline NMP concentration in MTAP^{prof} (red) and MTAP^{def} (blue) UC cell lines. Each cell line was retested 3 times. The symbol represents the mean along with SD bars. No significant difference was observed between the MTAP^{prof} and MTAP^{def} cell lines. N=3 biologically independent samples for each cell **(b)** Fold increase in NMPs upon treatment with pemetrexed (5 um). Statistical testing was done using Welch T test. No significant difference was observed between the MTAP^{prof} and MTAP^{def} cell lines.

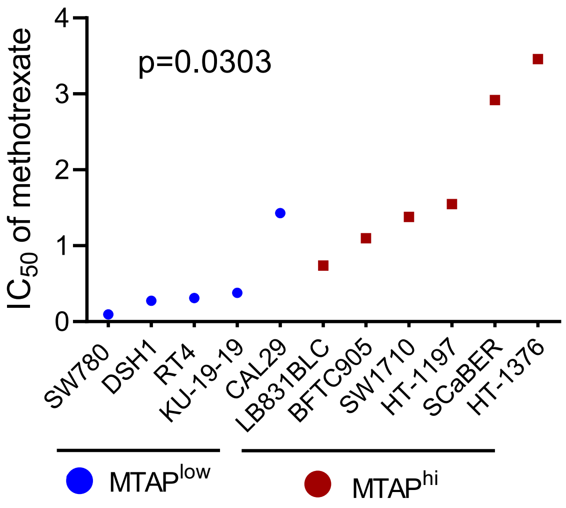

Supplementary Fig. 6. Analysis of the Genomics of Drug Sensitivity in Cancer (GDSC) dataset. $MTAP^{def}$, MTAP deficient; $MTAP^{prof}$, MTAP proficient; IC50, drug concentration that reduces viability by 50%. **(a)** GDSC analysis shows lower methotrexate IC50 for $MTAP^{def}$ compared $MTAP^{prof}$ UC cell lines. Statistical comparison was done using Mann-Whitney test.

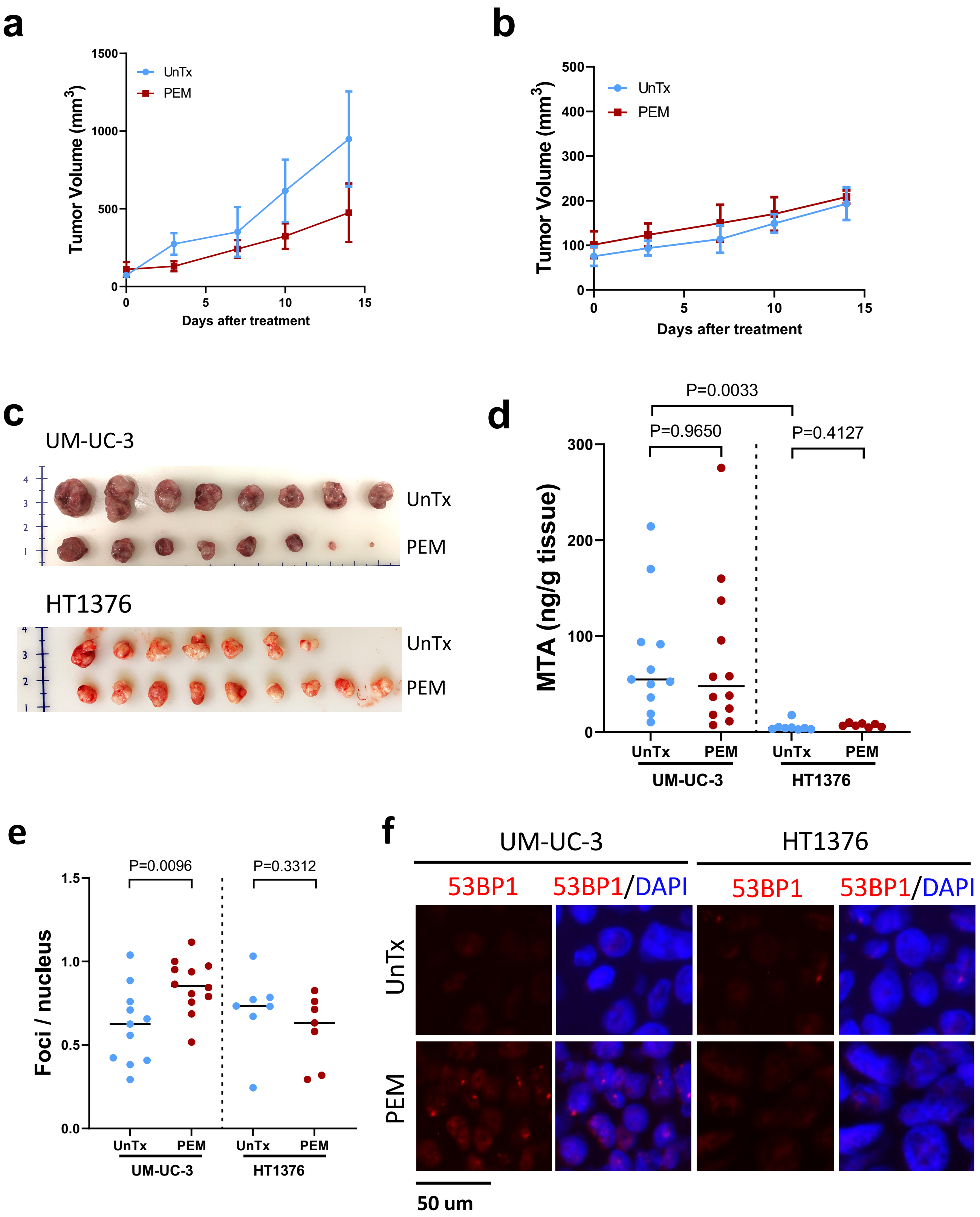

Supplementary Fig. 7. Effect of pemetrexed on MTAP^{def} tumor suppression is linked to DNA damage in xenograft mouse model. (a) and **(b)**, Tumor growth curves of pemetrexed (PEM) treated mice bearing UM- UC-3 **(a)** and HT1376 **(b)** tumors until two weeks after treatment. UC3 untreated (UnTx) n=11, UC3 PEM n=12, HT1376 UnTx n=7, HT1376 PEM n=7. Data are presented as mean +/- SD. **(c)** Image of tumors harvested from UM- UC-3 and HT1376. In UM-UC-3 mouse model, 3 untreated and 3 treated were collected early due to tumor ulceration; therefore, were not included in the image. **(d)** MTA levels detected in xenograft tumor tissues. Data were analyzed with two-sided Student's t test. **(e)** Quantitation of 53BP1 staining in xenograft tumor tissues, which was represented as 53BP1 foci per nucleus via ImageJ. Data were analyzed with two-sided Student's t test. **(f)** Representative images for 53BP1 immunofluorescent staining on xenograft tumor tissue FFPE slides.

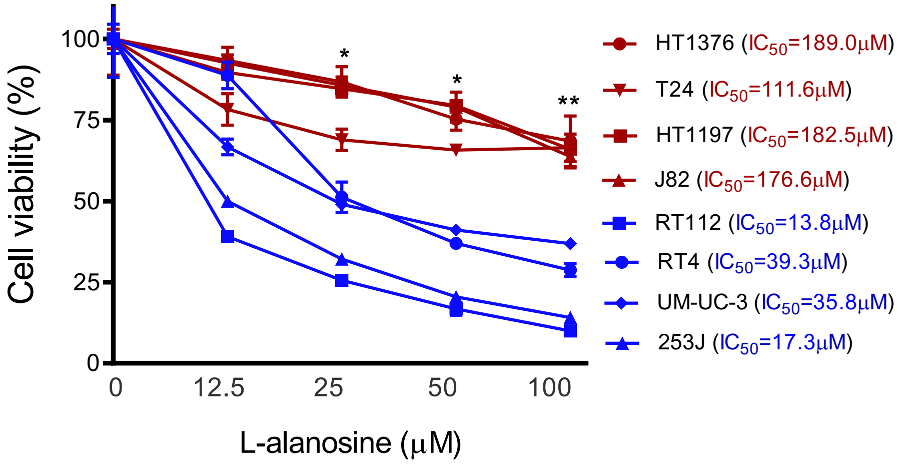

Supplementary Fig. 8. L-alanosine effect on cell viability among UC cell lines. **(a)** MTAP^{prof} UC cell lines are resistant to *de novo* adenine synthesis inhibitor L-alanosine. MTAP^{def} UC cell lines are sensitive to L- alanosine. Drug concentration that reduces viability by 50% (IC50) are displayed in the right upper corner. n=3 biologically independent samples for each cell line. Data are presented as mean +/- SD. Comparison was done using two-way ANOVA with Sidak's multiple comparisons test relative to solvent groups. *, p<0.05 and **, p<0.01.

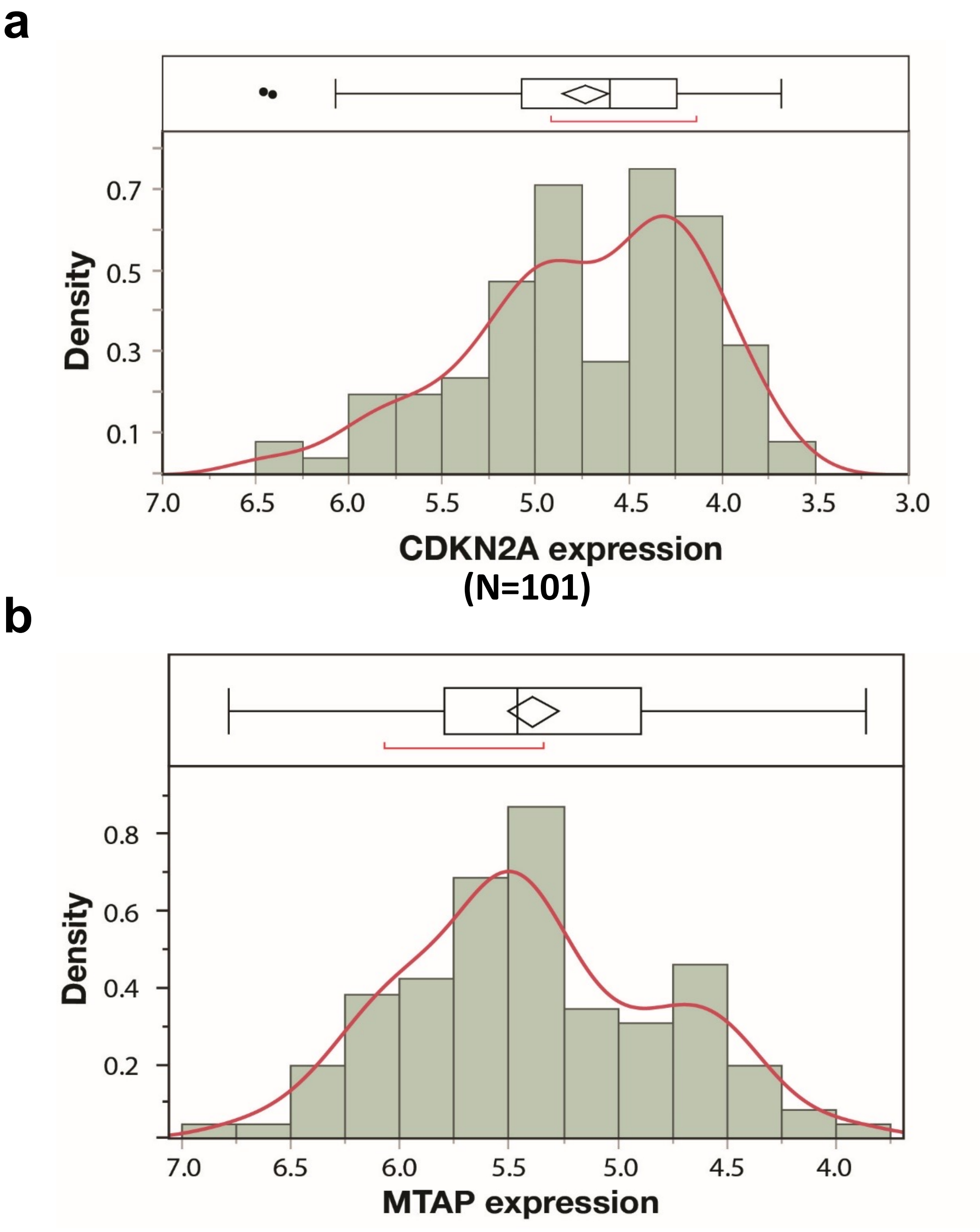

Supplementary Fig. 9. Distribution of *CDKN2A* and *MTAP* RNA expression in RNA Affymetrix microarray in lung adenocarcinoma. (a) *CDKN2A* has bimodal distribution around the median value of 4.4 (n = 101). **(b)** *MTAP* has bimodal distribution around the median value of 5.1 (n = 105). IQR = the 3rd quartile minus the 1st quartile. Whiskers drawn to the furthest point within 1.5 x IQR from the box. Potential outliers (disconnected points). A red bracket defining the shortest half of the data (the densest region).

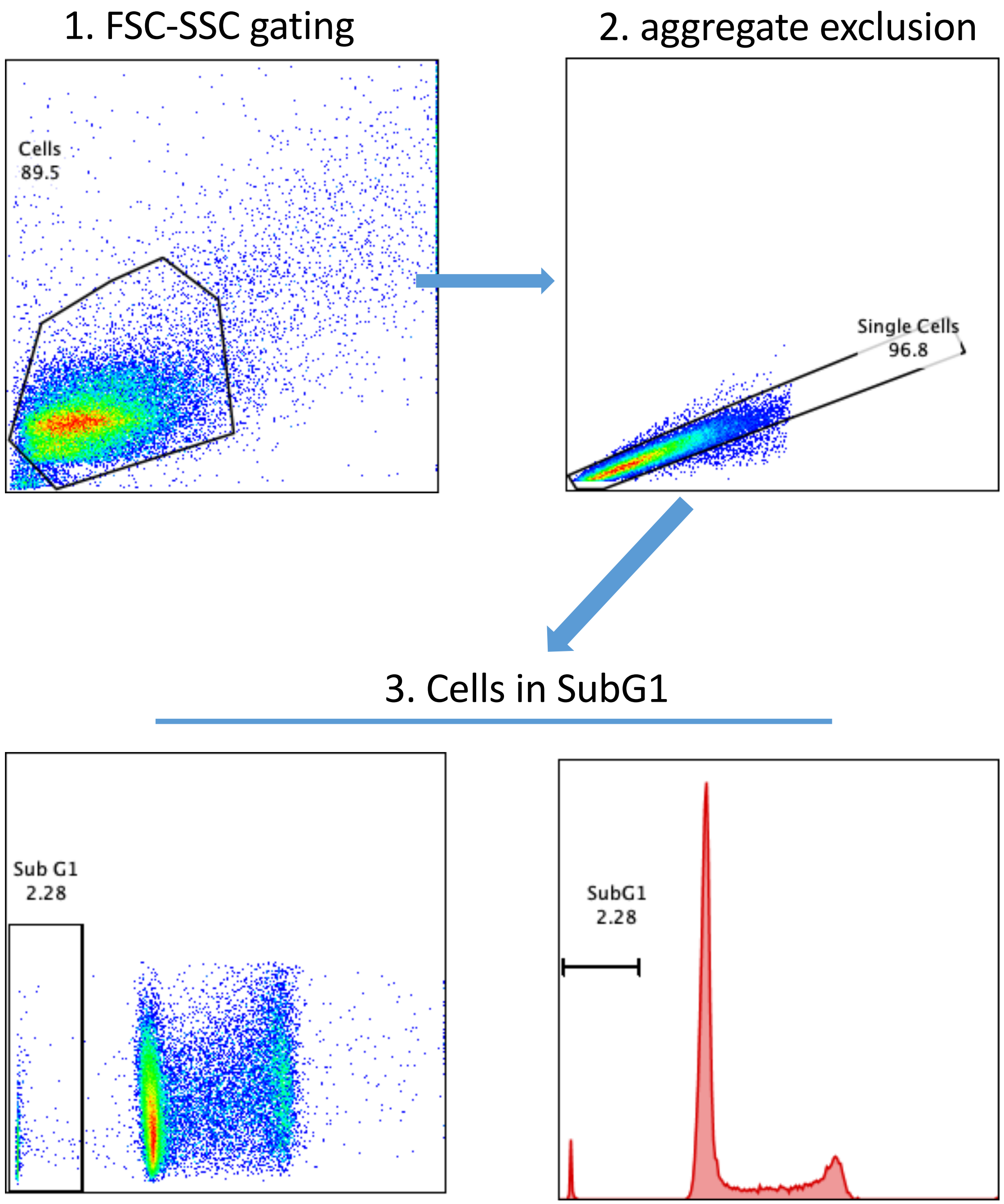

Supplementary fig. 10. Gating strategy for SubG1. Artifact exclusion included cell morphology (FSC-SSC) gating (1), aggregate exclusion (2), and propidium iodide (PI) gating for cells in SubG1 (3)

Supplementary Table 1. MTAP protein deficiency rate per bladder tumor tissue microarray IHC

Tumor stage	**$MTAP^{def}$**	**$MTAP^{prof}$**	**Sum**
Tis-T1	12(24.5%)	37(75.5%)	**49**
T2-4a	15(24.6%)	46(75.4%)	**61**
LN+	4 (40%)	6(60%)	**10**
M+	11(35.5%)	20 (64.5%)	**31**
Total	**42(27.8%)**	**109(72.2%)**	**151**

$MTAP^{def}$: MTAP deficient, $MTAP^{prof}$: MTAP proficient. Tis-T1: noninvasive, including Tis (in situ), Ta, and T1; T2-4a: invasive, including tumors invasive into muscularis propria, fat, prostate, and seminal vesicle; LN+: invasive to lymph nodes; M+: tumor metastasized to other organs. No difference was detected between T1 and other groups with two-tailed Fisher’s exact test.

Supplementary Table 2: All Treatment Related Adverse Events

	Total (N=7)			
	All grades		Grade ≥3	
All Treatment Related Adverse Event	**N**	**(%)**	**N**	**(%)**
**Any Event**	7	100%	3	43%
**Anemia**	7	100%	3	43%
**Anorexia**	1	14%	0	0%
**Aspartate aminotransferase increased**	1	14%	0	0%
**Fatigue**	3	43%	0	0%
**Hallucinations**	1	14%	0	0%
**Hypertension**	1	14%	0	0%
**Lip infection**	1	14%	0	0%
**Metabolism and nutrition disorders - Other, specify**	1	14%	0	0%
**Mucositis oral**	1	14%	0	0%
**Nausea**	3	43%	0	0%
**Neutrophil count decreased**	1	14%	0	0%
**Rash maculo-papular**	1	14%	0	0%
**Skin hyperpigmentation**	1	14%	0	0%
**Skin infection**	1	14%	1	14%
**Somnolence**	1	14%	0	0%
**Vomiting**	2	29%	1	14%

Supplementary Table 3: All Adverse Events Regardless of Relation

	Total (N=7)		Total (N=7)	
	All grades		All grades	
All Adverse Events	N	(%)	N	(%)
**Any Event**	7	100%	6	86%
**Anemia**	7	100%	3	43%
**Pain**	5	71%	2	29%
**Creatinine increased**	6	86%	1	14%
**Fatigue**	6	86%	1	14%
**Hyperglycemia**	3	43%	1	14%
**Hematuria**	2	29%	1	14%
**Platelet count decreased**	2	29%	1	14%
**Vomiting**	2	29%	1	14%
**Blood bilirubin increased**	1	14%	1	14%
**Hypotension**	1	14%	1	14%
**Renal and urinary disorders - Other, specify**	1	14%	1	14%
**Sinus tachycardia**	1	14%	1	14%
**Skin infection**	1	14%	1	14%
**Thromboembolic event**	1	14%	1	14%
**Vaginal hemorrhage**	1	14%	1	14%
**Insomnia**	6	86%	0	0%
**Back pain**	5	71%	0	0%
**Constipation**	5	71%	0	0%
**Nausea**	5	71%	0	0%
**Allergic rhinitis**	4	57%	0	0%
**Anorexia**	4	57%	0	0%
**Cough**	4	57%	0	0%
**Gastroesophageal reflux disease**	4	57%	0	0%
**Hypertension**	4	57%	0	0%
**Hypothyroidism**	4	57%	0	0%
**Dyspnea**	3	43%	0	0%
**Urinary urgency**	3	43%	0	0%
**Cholesterol high**	2	29%	0	0%
**Edema limbs**	2	29%	0	0%
**Hypomagnesemia**	2	29%	0	0%
**Hyponatremia**	2	29%	0	0%
**Paresthesia**	2	29%	0	0%
**Alkaline phosphatase increased**	1	14%	0	0%

**Arthralgia**	1	14%	0	0%
**Arthritis**	1	14%	0	0%
**Aspartate aminotransferase increased**	1	14%	0	0%
**Bronchospasm**	1	14%	0	0%
**Depression**	1	14%	0	0%
**Diarrhea**	1	14%	0	0%
**Dizziness**	1	14%	0	0%
**Dry mouth**	1	14%	0	0%
**Dry skin**	1	14%	0	0%
**Fall**	1	14%	0	0%
**Fever**	1	14%	0	0%
**Flank pain**	1	14%	0	0%
**Glaucoma**	1	14%	0	0%
**Hallucinations**	1	14%	0	0%
**Headache**	1	14%	0	0%
**Hearing impaired**	1	14%	0	0%
**Hoarseness**	1	14%	0	0%
**Hypokalemia**	1	14%	0	0%
**Infections and infestations - Other, specify**	1	14%	0	0%
**Infusion site extravasation**	1	14%	0	0%
**Investigations - Other, specify**	1	14%	0	0%
**Lip infection**	1	14%	0	0%
**Localized edema**	1	14%	0	0%
**Lymphocyte count decreased**	1	14%	0	0%
**Metabolism and nutrition disorders - Other, specify**	1	14%	0	0%
**Mucositis oral**	1	14%	0	0%
**Musculoskeletal and connective tissue disorder - Other, specify**	1	14%	0	0%
**Neutrophil count decreased**	1	14%	0	0%
**Oral cavity fistula**	1	14%	0	0%
**Peripheral sensory neuropathy**	1	14%	0	0%
**Rash maculo-papular**	1	14%	0	0%
**Rectal pain**	1	14%	0	0%
**Respiratory, thoracic and mediastinal disorders - Other, specify**	1	14%	0	0%
**Sinusitis**	1	14%	0	0%
**Skin hyperpigmentation**	1	14%	0	0%
**Somnolence**	1	14%	0	0%
**Sore throat**	1	14%	0	0%

**Urinary frequency**	1	14%	0	0%
**Vaginal infection**	1	14%	0	0%
**Weight loss**	1	14%	0	0%
**White blood cell decreased**	1	14%	0	0%

Supplementary Table 4: Response of patients with lung adenocarcinoma to pemetrexed-containing chemotherapy based on CDKN2A/MTAP status by mRNA

	PD	SD ≥6 mos	SD <6 mos	PR	CR	ORR (N)	CB (N)
$CDKN2A^{hi}/MTAP^{hi}$ (n=25)	7	4	7	7	0	28% (7)	44% (11)
$CDKN2A^{hi}/MTAP^{lo}$(n=13)	3	4	2	4	0	31% (4)	61% (8)
$CDKN2A^{lo}/MTAP^{hi}$ (n=8)	3	5	0	0	0	0% (0)	62% (5)
$CDKN2A^{lo}/MTAP^{lo}$ (n=26)	5	1	6	13	1	54% (14)	58% (15)
Total (n=72)	18	14	15	24	1	35% (25)	54% (39)

PD: progressive disease, SD: stable disease, mos: months, PR: partial response, CR: complete response, ORR: overall response rate, CB: clinical benefit. ORR was defined as PR + CR. CB was defined as SD ≥6 mos + CR + PR.

Supplementary Table 5: generalized linear model (GLM) estimating the odds ratio and p-value for most frequently altered genes in the lung cancer cohort (n=72). We estimate the correlation of 10 most altered genes in lung cancer beside MTAP to estimate the odds ratio and p-value for each gene independently. Genes with an odds ratio >1 (log (odds ratio) >0) and a p-value <0.05 are considered to be positively correlated with response. Genes with an odds ratio <1 (log (odds ratio) <0) and a p-value <0.05 are considered to be negatively correlated with response. Adjustments were made for multiple gene comparisons and false discovery rate and presented as q values.

geneName	OddsRatio	coeff	cilow	cihigh	p_value	q_value
MTAP	0.392732231	-0.934627244	0.163167375	0.875693009	0.027502371	0.302526078
PIK3CA	0.396821832	-0.924267886	0.142228169	0.991731621	0.05927728	0.326025038
CDKN2A	0.630573777	-0.461125117	0.267128178	1.395751323	0.26886466	0.475911601
TP53	0.948629464	-0.052737006	0.456917359	1.979880005	0.886436851	0.886436851
ROS1	1.143344857	0.133958052	0.885756175	1.481448888	0.302852837	0.475911601
RET	1.148192416	0.138188894	0.26788913	4.591067475	0.845135547	0.886436851
MET	1.226373229	0.20406122	0.921204317	1.661766255	0.169596079	0.373111374
KRAS	1.263453703	0.233849005	0.500967174	3.241732772	0.619256902	0.85147824
ALK	1.402106297	0.337975604	0.068735069	30.3297377	0.825786859	0.886436851
EGFR	1.620237041	0.48257246	0.858411649	3.231309557	0.148775108	0.373111374
BRAF	2.317219518	0.840367983	0.725596731	8.220870683	0.167996811	0.373111374
