# Supplementary material for: MTAP deficiency creates an exploitable target for antifolate therapy in 9p21-loss cancers
Source: Nat Commun. 2022 Apr 4;13:1797. doi: 10.1038/s41467-022-29397-z (PMC8980015; doi:10.1038/s41467-022-29397-z)
Supplement: Supplementary file 3 — Reporting summary [file 41467_2022_29397_MOESM3_ESM.pdf]

nature research

Corresponding author(s): Linghua Wang; Jianjun Gao

Last updated by author(s): 01/30/2022

Reporting Summary

Nature Research wishes to improve the reproducibility of the work that we publish. This form provides structure for consistency and transparency in reporting. For further information on Nature Research policies, see our Editorial Policies and the Editorial Policy Checklist.

Statistics

For all statistical analyses, confirm that the following items are present in the figure legend, table legend, main text, or Methods section.

n/a	Confirmed	
☐	☒	The exact sample size (n) for each experimental group/condition, given as a discrete number and unit of measurement
☐	☒	A statement on whether measurements were taken from distinct samples or whether the same sample was measured repeatedly
☐	☒	The statistical test(s) used AND whether they are one- or two-sided<br>*Only common tests should be described solely by name; describe more complex techniques in the Methods section.*
☐	☒	A description of all covariates tested
☐	☒	A description of any assumptions or corrections, such as tests of normality and adjustment for multiple comparisons
☐	☒	A full description of the statistical parameters including central tendency (e.g. means) or other basic estimates (e.g. regression coefficient) AND variation (e.g. standard deviation) or associated estimates of uncertainty (e.g. confidence intervals)
☐	☒	For null hypothesis testing, the test statistic (e.g. F, t, r) with confidence intervals, effect sizes, degrees of freedom and P value noted<br>*Give P values as exact values whenever suitable.*
☒	☐	For Bayesian analysis, information on the choice of priors and Markov chain Monte Carlo settings
☒	☐	For hierarchical and complex designs, identification of the appropriate level for tests and full reporting of outcomes
☒	☐	Estimates of effect sizes (e.g. Cohen's d, Pearson's r), indicating how they were calculated

Our web collection on statistics for biologists contains articles on many of the points above.

Software and code

Policy information about availability of computer code

Data collection	SAS 9.4 (The SAS Institute Inc, Cary, NC), Stata 14.1 (StatCorp, College Station, TX). Graphpad Prism 7.0 (Graphpad Software, San Diego, CA), MassHunter software (Agilent, Santa Clara, CA).
Data analysis	SAS 9.4 (The SAS Institute Inc, Cary, NC), Stata 14.1 (StatCorp, College Station, TX). Graphpad Prism 7.0 (Graphpad Software, San Diego, CA), MassHunter software (Agilent, Santa Clara, CA).

For manuscripts utilizing custom algorithms or software that are central to the research but not yet described in published literature, software must be made available to editors and reviewers. We strongly encourage code deposition in a community repository (e.g. GitHub). See the Nature Research guidelines for submitting code & software for further information.

Data

Policy information about availability of data

All manuscripts must include a data availability statement. This statement should provide the following information, where applicable:

- Accession codes, unique identifiers, or web links for publicly available datasets
- A list of figures that have associated raw data
- A description of any restrictions on data availability

The data generated or analyzed during this study are included in this published article, its Supplementary Information and Source Data files. For TCGA BLCA cohort shown in Fig 1b, the genomic data can be retrieved from NCI Genomic Data Commons (NCI-GDC: https://gdc.cancer.gov). Publicly available datasets pertaining to Supp Fig 2a can be downloaded from https://depmap.org/portal/download/. Tissue microarray data pertaining to Fig 1c,d is included in Source Data file. Clinical data pertaining Fig 1e, g, h is included in Source Data file. Uncropped Western Blot gels and in vitro data pertaining to Fig 2a-g is included in Source Data file. Clinical data and gene expression data pertaining to Fig 4a-e is included in Source Data file. Additional data related to the current study are available from the corresponding author (Jianjun Gao) on reasonable request that does not include confidential patient information.

April 2020

Field-specific reporting

Please select the one below that is the best fit for your research. If you are not sure, read the appropriate sections before making your selection.

☒ Life sciences ☐ Behavioural & social sciences ☐ Ecological, evolutionary & environmental sciences

For a reference copy of the document with all sections, see nature.com/documents/nr-reporting-summary-flat.pdf

Life sciences study design

All studies must disclose on these points even when the disclosure is negative.

Sample size	Sample size for NCT02693717: a maximum of 25 MTAP deficient patients were planned to be enrolled from MD Anderson at an estimated accrual rate of 1-2 patients per month. The sample size of 25 patients allows that if the trial continues to full size and has a response rate of 40% for pemetrexed, the Bayesian posterior 95% credible interval will be (22%, 58%) using a prior of beta (0.56, 1.44). Interim monitoring for futility and efficacy will be performed once 10 patients are evaluable, with continuing enrollment during analyses. After the first analysis, the trial will be monitored every 2 months with continuous entry. If the true response rate is 40%, the trial will stop early 14% of the time. If it's very bad, like 20%, the trial will stop 79% of the time, and if it's as high as 60% it will stop less than 1% of the time.<br><br>In vitro and in vivo sample sizes were chosen based on similar studies from the literature and were large enough to detect statistically significant differences between groups. please see doi:10.3390/cancers12010010 and doi:10.1038/bjc.2013.216. Exact sample sizes are indicated in the figure legends.
Data exclusions	For urothelial cancer data, patients who received pemetrexed but did not have tissue available for MTAP staining or did not have available staging imaging to assess response were excluded. Regarding lung cancer data, patients with squamous cell histology, patients who did not have RNA expression performed, or patients who did not have available staging imaging to assess response were excluded.
Replication	In vitro experiments for determination of cell cycle distribution by flow cytometery after treatment with pemetrexed were repeated at least three times. Replication was successful.
Randomization	Allocation was not random as this was a single arm study.
Blinding	Blinding is not relevant to our study as this was a single arm study. All in vitro and animal studies were planned and performed by the same investigator to ensure that each experiment contains all groups and appropriate controls. Investigators were blinded to group allocation during data collection and/or analysis.

Reporting for specific materials, systems and methods

We require information from authors about some types of materials, experimental systems and methods used in many studies. Here, indicate whether each material, system or method listed is relevant to your study. If you are not sure if a list item applies to your research, read the appropriate section before selecting a response.

Materials & experimental systems

n/a	Involved in the study	
☐	☒	Antibodies
☐	☒	Eukaryotic cell lines
☒	☐	Palaeontology and archaeology
☐	☒	Animals and other organisms
☐	☒	Human research participants
☐	☒	Clinical data
☒	☐	Dual use research of concern

Methods

n/a	Involved in the study	
☒	☐	ChIP-seq
☐	☒	Flow cytometry
☒	☐	MRI-based neuroimaging

Antibodies

Antibodies used	Western blotting: anti-MTAP polyclonal (11475-1-AP, ProteinTech); anti-poly (ADP-ribose) polymerase-1 (PARP-1), clone 46D11 (9532, Cell Signaling); anti-p16 INK4A, clone D7C1M (80772, Cell Signaling); and anti-B-actin, clone AC-40 (A3853, MilliporeSigma); goat-anti-mouse IgG-HRP (sc-2005, Santa Cruz); goat-anti-rabbit IgG-HRP (sc-2004, Santa Cruz).<br>Immunofluorencent staining: anti-r-H2AX clone GT2311 (MA5-27753, ThermoFisher); anti-53BP1 polyclonal (4937, Cell Signaling); goat anti-rabbit IgG AF594 (A-11012, ThermoFisher); goat anti-mouse IgG AF594 (A-11020, ThermoFisher).
Validation	Only commercially available antibodies were used in this study. These antibodies are well-established and validation was carried out by the manufacturers as stated on the manufacturers website.

Eukaryotic cell lines

Policy information about cell lines

Cell line source(s)	All cell lines were originally obtained from ATCC (Manassas, VA), except for RT112 from Creative Bioarray (Shirley, NY). HT-1376, HT-1197, J82, and UM-UC-3 were maintained in MEM supplemented with 10% FBS, 2 mM L-glutamine, and 0.1 mM non-essential amino acids. 253J was maintained in DMEM plus 10% FBS. RT112 was maintained in RPMI1640 plus 10%FBS. T24 and RT4 were grown in McCoy's 5A plus 10% FBS.
Authentication	None of the cell lines used were authenticated.
Mycoplasma contamination	Only mycoplasma-free cultures were used.
Commonly misidentified lines (See ICLAC register)	No misidentified lines were used

Animals and other organisms

Policy information about studies involving animals; ARRIVE guidelines recommended for reporting animal research

Laboratory animals	6 weeks old male athymic nude mice were purchased from the Charles River Laboratory. Housing details are provided in Methods.
Wild animals	study did not involve wild animals
Field-collected samples	study did not involve samples collected from the field
Ethics oversight	Animal experiments were carried out under conditions adhering to approved protocols from the Institutional Animal Care and Use Committee at the University of Texas MD Anderson Cancer Center.

Note that full information on the approval of the study protocol must also be provided in the manuscript.

Human research participants

Policy information about studies involving human research participants

Population characteristics	NCT02693717 is a single-arm phase II clinical trial to evaluate pemetrexed disodium in previously treated metastatic MTAP deficient urothelial cancer. Patients were required to have histological confirmation of metastatic UC and sufficient tumor tissues for MTAP IHC testing. Patients who had received any non-antifolate-containing systemic therapy (including immunotherapy) were eligible.<br><br>Seven patients enrolled on NCT02693717. Median age (min, max) was 71 (68, 80). F:M ratio was 4:3. ECOG performance status was 0 and 1 in 3 and 4 patients, respectively. All patients had prior systemic therapy for urothelial cancer. All patients were MTAP deficient on IHC.<br><br>Seventy-two patients were included from the BATTLE2 lung adenocarcinoma cohort. Characteristics for the MTAPlo/CDKN2Alo group (N=26) include: median age (min,max) of 60.5 (34, 82), F:M ratio of 2:1, EGFR mutation rate of 42%. 62% had not received prior systemic therapy. Characteristics for the rest of the cohort (N=46) include: median age (min,max) of 59 (26, 76), F:M ratio of 1:1, EGFR mutation rate of 16%. 70% had not received prior systemic therapy.
Recruitment	NCT02693717: Patients were recruited by medical oncologist who treat urothelial cancer at MD Anderson. The total estimated accrual for this trial was 25 patients. However, only seven patients were enrolled due to competing protocols and the opening of NCT03744793. Competing trials might lead to self-selection leading to smaller sample size and decreased power.<br><br>Patient samples for tissue microarray IHC: patients eligible for enrollment were those seen at Mayo Clinic (Scottsdale, Arizona) who were > 18 years old, able to provide informed consent, and undergoing evaluation for genitourinary diseases. Patients were contacted during routine clinical visits or in preoperative settings within Mayo Clinic departments and divisions, including urology, radiation oncology, pathology, and medical oncology. Patients were excluded if they declined to participate or if the banking of their biospecimens would compromise the availability of tissue for diagnosis and standard clinical care. The protocol for collecting biospecimens, the process for consenting patients, and the current informed consent form were approved by the Mayo Clinic IRB (protocol no. 08-000980). Patients were enrolled from June 1, 2010, through January 1, 2013.
Ethics oversight	NCT02693717 was approved by the Institutional Review Boards (IRB) of MDACC (MDACC protocol 2015-0592). Urothelial cancer historic cohort was approved by the MDACC IRB under PA17-0577. Patient samples for tissue microarray IHC were approved by the Mayo Clinic IRB (protocol no. 08-000980). The lung cancer cohort were approved by the MDACC IRB under the BATTLE2 trial (MDACC protocol 2009-0360)

Note that full information on the approval of the study protocol must also be provided in the manuscript.

Clinical data

Policy information about clinical studies

All manuscripts should comply with the ICMJEguidelines for publication of clinical research and a completedCONSORT checklist must be included with all submissions.

Clinical trial registration	NCT02693717
Study protocol	Full trial protocol has been shared with the editor
Data collection	Safety data regarding therapy were collected every 3 weeks during clinic return visits. Response assessment data were collected every 3 months during staging procedures. Subsequent pemetrexed treatment may be carried out at the MDACC local Regional Cancer Centers or local physician office. However, initial screening, workup, the first dose of pemetrexed, restaging, and biological sample collection must be done at the MDACC main campus. Patients with local physicians who agree to manage standard infusions must agree to perform required tests, send all related medical records to MDACC, and allow the MDACC investigator to direct dose adjustments.Enrollement was done between 9/1/2017 and 1/25/2019.
Outcomes	The primary objective of this trial was to evaluate the RR defined by Response Evaluation Criteria in Solid Tumors (RECIST) 1.1. The secondary objectives included: 1) Progression free survival (PFS) and 2) overall survival (OS). Response was considered to be complete (CR) or partial (PR) per RECIST 1.1. The primary outcome measures were performed by the principle investigator and confirmed by another investigator who was blinded to the initial assessment. All other responses, including not evaluated were considered non-responses. AEs graded according to the National Cancer Institute Common Terminology Criteria for Adverse Events (CTCAE) version 4.03. OS was defined as the time from the first day of pemetrexed treatment until death or last contact. PFS was defined as the time from the first day of pemetrexed treatment until disease progression, death, or last disease assessment. Patients alive and without progression at their last assessment were censored on their last date of assessment before starting a new treatment. Kaplan-Meier curves are presented for OS, and PFS.

Flow Cytometry

Plots

Confirm that:

- [x] The axis labels state the marker and fluorochrome used (e.g. CD4-FITC).
- [x] The axis scales are clearly visible. Include numbers along axes only for bottom left plot of group (a 'group' is an analysis of identical markers).
- [x] All plots are contour plots with outliers or pseudocolor plots.
- [x] A numerical value for number of cells or percentage (with statistics) is provided.

Methodology

Sample preparation	After treatment for designated time, cells were collected and adjusted to a concentration of 10^5 cells/mL, fixed by 75% ethanol for more than 1h, and then treated with RNase A for 30 min at room temperature. After 1 hour incubation with PI staining, samples were used for FACS analysis.
Instrument	BD Bioscience FACS Canto II
Software	Data were collected with BD FACS Diva v6.0 and analyzed with FlowJo v10.
Cell population abundance	For each sample, cells were collected from cell line culture which contained pure cell population. At least 30k events were collected after aggregation exclusion.
Gating strategy	Gating strategy for SubG1. Artifact exclusion included cell morphology (FSC-SSC) gating, aggregate exclusion, and propidium iodide (PI) gating for cells in SubG1.

- [x] Tick this box to confirm that a figure exemplifying the gating strategy is provided in the Supplementary Information.
